# Secondary Somatic Mutations in G-Protein-Related Pathways and Mutation Signatures in Uveal Melanoma

**DOI:** 10.3390/cancers11111688

**Published:** 2019-10-30

**Authors:** Francesca Piaggio, Veronica Tozzo, Cinzia Bernardi, Michela Croce, Roberto Puzone, Silvia Viaggi, Serena Patrone, Annalisa Barla, Domenico Coviello, Martine J. Jager, Pieter A. van der Velden, Michael Zeschnigk, Davide Cangelosi, Alessandra Eva, Ulrich Pfeffer, Adriana Amaro

**Affiliations:** 1 Tumor Epigenetics; IRCCS Ospedale Policlinico San Martino, 16132 Genova, Italy; francesca.piaggio@hsanmartino.it (F.P.); cinzia.bernardi@yahoo.it (C.B.); adriana.amaro@hsanmartino.it (A.A.); 2 DIBRIS, University of Genova, 16145 Genova, Italy; veronica.tozzo@dibris.unige.it (V.T.); annalisa.barla@unige.it (A.B.); 3 Biotherapy; IRCCS Ospedale Policlinico San Martino, 16132 Genova, Italy; michela.croce@hsanmartino.it;; 4 Clinical Epidemiology, IRCCS Ospedale Policlinico San Martino, 16132 Genova, Italy; roberto.puzone@hsanmartino.it; 5 DISTAV, University of Genova, 16132 Genova, Italy; silvia.viaggi@unige.it; 6 IRCCS Istituto G. Gaslini, 16147 Genova, Italy; 3040005@studenti.unige.it (S.P.); coviello@unige.it (D.C.); 7 Laboratory of Human Genetics, Department of Ophthalmology, Leiden University Medical Center, 2333 ZA Leiden, The Netherlands; M.J.Jager@lumc.nl (M.J.J.); P.A.van_der_Velden@lumc.nl (P.A.v.d.V.); 8 Institute of Human Genetics, University Clinics Essen, University Duisburg-Essen, 45147 Essen, Germany; Michael.zeschnigk@uni-due.de; 9 Laboratory of Molecular Biology, IRCCS Istituto Giannina Gaslini, 16147 Genova, Italy; davide.cangelosi@gmail.com (D.C.); alessandraeva@gaslini.org (A.E.)

**Keywords:** driver mutation, gene set enrichment, tumor evolution, mutation signature

## Abstract

Background: Uveal melanoma (UM), a rare cancer of the eye, is characterized by initiating mutations in the genes G-protein subunit alpha Q (*GNAQ*), G-protein subunit alpha 11 (*GNA11*), cysteinyl leukotriene receptor 2 (*CYSLTR2*), and phospholipase C beta 4 (*PLCB4*) and by metastasis-promoting mutations in the genes splicing factor 3B1 (*SF3B1*), serine and arginine rich splicing factor 2 (SRSF2), and BRCA1-associated protein 1 (*BAP1*). Here, we tested the hypothesis that additional mutations, though occurring in only a few cases (“secondary drivers”), might influence tumor development. Methods: We analyzed all the 4125 mutations detected in exome sequencing datasets, comprising a total of 139 Ums, and tested the enrichment of secondary drivers in Kyoto Encyclopedia of Genes and Genomes (KEGG) pathways that also contained the initiating mutations. We searched for additional mutations in the putative secondary driver gene protein tyrosine kinase 2 beta (*PTK2B*) and we developed new mutational signatures that explain the mutational pattern observed in UM. Results: Secondary drivers were significantly enriched in KEGG pathways that also contained *GNAQ* and *GNA11*, such as the calcium-signaling pathway. Many of the secondary drivers were known cancer driver genes and were strongly associated with metastasis and survival. We identified additional mutations in *PTK2B*. Sparse dictionary learning allowed for the identification of mutational signatures specific for UM. Conclusions: A considerable part of rare mutations that occur in addition to known driver mutations are likely to affect tumor development and progression.

## 1. Introduction

Uveal melanoma (UM) is a rare tumor with an incidence of 0.2–0.8/100,000 per year. Diagnosis of UM is made by ophthalmoscopic examination. Treatment options are local radiotherapy, enucleation, and endoresection, all of which provide an effective treatment of the primary tumor. Nonetheless, almost half of patients will develop distant metastases with the liver as the first and major target organ. The 5 year survival rate is 69%. Median survival after development of clinical metastases is less than a year. There are no adjuvant treatments available and essentially no efficacious therapies for metastatic disease (for recent reviews see [1,2,3]). 

UM is molecularly distinct from cutaneous melanoma (CM)—the two diseases are characterized by completely different driver mutations, B-Raf proto-oncogene (*BRAF*), *NRAS* proto-oncogene (*NRAS*), and neurofibromin 1 (*NF1*) [4] in CM and G-protein subunit alpha Q (*GNAQ*) [5], G-protein subunit alpha 11 (*GNA11*) [6], cysteinyl leukotriene receptor 2 (*CYSLTR2*), phospholipase C beta 4 (*PLCB4*) [7], *BRCA1*-associated protein 1 (*BAP1*) [5,6,8], splicing factor 3B1 (*SF3B1*) [9], serine and arginine rich splicing factor 2 (*SRSF2*) [10], and eukaryotic translation initiation factor 1A X-linked (*EIF1AX*) [11] in UM. The highly conserved genes *GNAQ* and *GNA11* show two hotspot mutations in identical positions (R183 and Q209) that occur in at least 80% of all UM cases [3,12,13]. *GNAQ* and *GNA11* encode G-protein alpha subunits and the mutations inactivate the guanosine triphosphate hydrolase (GTP-ase) function of the proteins that remain in an active, signal transducing conformation [5,6]. CYSLTR2 is activated by endogenous leukotrienes and transmits intracellular signals through GNAQ and GNA11 [14]. It carries activating mutations in 3% of UM [3]. PLCB4 carries activating mutations [7] in 4% of UM [3] and catalyzes the formation of inositol 1,4,5-trisphosphate and diacylglycerol from phosphatidylinositol 4,5-bisphosphate using calcium as a cofactor. PLCB4 acts downstream of GNAQ and GNA11 and plays an important role in intracellular signal transduction [7]. GNAQ, GNA11, CYSLTR2, and PLCB4 are considered as initiating mutations that occur in a mutually exclusive manner and account for 85%–90% of UM cases [3]. BAP1 [8], SF3B1 [9], and SRSF2 [10] mutations are linked to tumor progression and usually occur in concomitance to the initiating mutations. EIF1AX usually occurs in concomitance to one of the four initiating mutations and is not associated with tumor progression [11]. 

UM shows a very low mutational burden with a mean, according to the mutation algorithms applied, of only 17 [15] to 30 [10] non-synonymous mutations in protein coding sequences per exome or 0.5 mutations per megabase [7]. UM and CM [15] show similar mutation patterns that are dominated by C>T transitions. In CM, these transitions occur in the context of TCN (where the central C is the actually mutated nucleotide and “N” indicates any nucleotide) which is part of Alexandrov’s signature n.7 and is likely due to ultraviolet radiation [16,17]. In UM, C>T transitions prevalently occur in the context of CCN. Despite evidence that UV-light exposure is a risk factor for UM [18,19], the UV-associated signature is not observed in UM. 

Field and co-workers performed a thorough re-analysis of next generation sequencing data from three cohorts for a total of 139 UM cases [10]. This analysis revealed an evolutionary path of UM that is best described as punctuated equilibrium, a concept adopted from evolution theory [20,21] that describes a burst of genomic alterations followed by linear outgrowth of genomically stable clones. Following this interpretation, UM mutations (including the metastasis-associated BAP1 mutation) occur early during UM tumorigenesis. The initial mutational burst creates a template of cells carrying multiple mutations, some of which are selected for growth during tumor evolution. 

We address here the question of whether other somatic mutations that occur concomitantly with the four putative initiating mutations or during tumor growth show some enrichment for specific pathways, indicative of a co-driver function, or whether they occur completely at random as simple bystander mutations. This question, that might be relevant for all tumor types, can be easily addressed for UM because an enrichment in a specific pathway can more easily be documented among the few mutations typical of UM. This aspect has not been addressed by previous reports on massive parallel sequencing of UM cases [8,9,10,11,22]. We also apply a novel approach to the identification of mutational signatures on UM and CM to dissect possible differences in the profiles of the two tumors. We furthermore describe additional mutations in the protein tyrosine kinase 2 beta (*PTK2B*) gene, suggesting its function as a putative co-driver gene.

## 2. Results

### 2.1. Enrichment of Secondary Mutations in G-Protein Related Pathways

We collected all mutations from the 139 cases of the combined re-analysis performed by Field et al. [10] which includes the The Cancer Genome Atlas (TCGA) UM dataset of 80 cases [15], the Essen dataset of 22 cases [11], and the Miami dataset of 37 cases [9]. Two unusual cases from the TCGA dataset were included in the analysis: one case with the unusually high number of 222 non-synonymous mutations having R183S mutations in both *GNAQ* and *GNA11*, whereas another has a stop gain mutation in *GNAQ*. The 139 cases analyzed contained non-synonymous mutations in a total of 4125 genes (Table S1). The known UM driver mutations were recurrent (*GNAQ n* = 67, *GNA11 n* = 64, *CYSLTR2 n* = 7, *PLCB4 n* = 3, *BAP1 n* = 58, *SF3B1 n* = 31, *EIF1AX n* = 18, *SRSF2 n* = 3). 

Mutations in *GNAQ*, *GNA11*, *CYSLTR2*, and *PLCB4* are considered as potential initiating mutations because they are present in most if not all the cells of the tumors, as the mutations lead to constitutive activation of G-protein/calcium signaling [5,6,7,14] and because they are already present in nevi [23]. These mutations are, with some exceptions, mutually exclusive. In addition to these initiating mutations, there are frequent mutations in *BAP1* [8], *SF3B1* [9], and *SRSF2* [10] that promote metastasis, and in *EIF1AX* [11] that does not affect progression. In the following, we report on all the other mutations that occur infrequently and have so far been considered as bystander or passenger mutations [24]. 

In order to identify functional annotations for *GNAQ*, *GNA11*, *CYSLTR2*, and *PLCB4*, we interrogated the KEGG pathway database (https://www.genome.jp/kegg/pathway.html). GNAQ yielded 43 entries, *GNA11* 17, *CYSLTR2* 2, and *PLCB* 20 (PLCB4 yielded none). All annotations of *GNA11* and the majority of those of *PLCB* shared with those of *GNAQ*. The only annotation shared by all four genes was “calcium signaling pathway” (Table S2).

**Figure 1:**
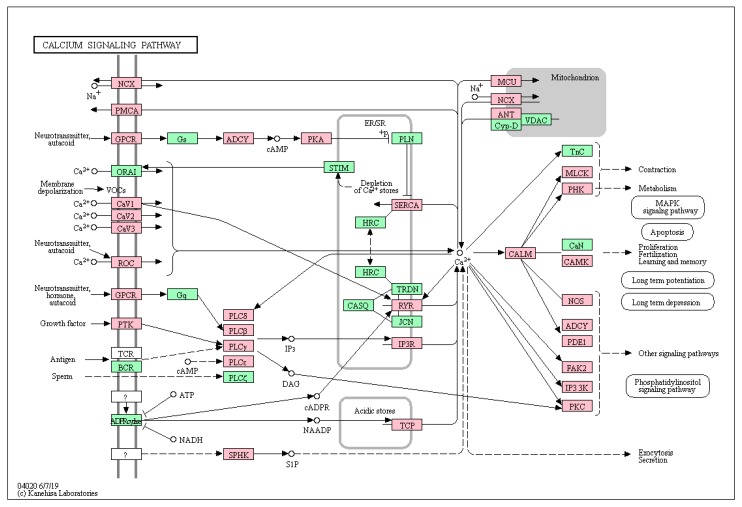
The KEGG calcium signaling pathway. Genes of the calcium pathway that carry a mutation in at least one case of uveal melanoma are indicated in red.

Of the 4117 genes carrying non-synonymous mutations (in the following termed “secondary mutations”, in contrast with the known driver mutations), 66 were annotated to belong to the calcium signaling pathway that contains 188 genes of the approximately 22,000 of the whole genome. This enrichment was highly significant (*p* = 0.0004) (Table 1). These mutations affected almost all the nodes of the pathway (Figure 1).

Eight other KEGG pathways were significantly enriched (adjusted *p*-value < 0.01) for annotations of the 4117 secondary mutation genes, three of which (aldosterone synthesis and secretion, Ras-proximate-1 (*RAP1*) signaling pathway, circadian entrainment) also contained *GNAQ* and *GNA11* (marked by an asterisk in Table 1). A total of 138 genes were annotated significant pathways that also contained *GNAQ* or *GNA11* (Table 1). A total of 14 additional pathways, containing “pathways in cancer”, “inositol phosphate metabolism”, and “melanogenesis”, were enriched considering the adjusted *p*-value of 0.05 (Table S3).

We then analyzed whether the secondary mutations can be considered driver mutations following the 20/20 rule, established by Vogelstein et al., that requires that at least 20% of all mutations encountered in cancers are hotspot mutations to classify the gene as an oncogene and truncating mutations to establish tumor suppressor function [25]. A total of 28 mutations could be classified as oncogenic and 31 as tumor suppressor mutations, 7 of which possessed both potential activities (Table S1). Because known oncogenes and tumor suppressor genes are also exposed to bystander mutations, only known hotspot mutations can be taken as evidence for potential oncogenic effects and protein truncating mutations as evidence for tumor suppressor function in the case of biallelic mutation (except for breast cancer associated 1 and 2 (BRCA1, BRACA2) for which gene dosage effects have been shown [26]). 

The oncogene isocitrate dehydrogenase (nicotinamide adenine diphosphate, NADP(+)) 1 (*IDH1*) carried the mutation R132C that has been observed in 1290 samples registered in the COSMIC database [27] and a total of 9829 samples showed an amino acid substitution in this position. The most frequent mutation affecting this amino acid (395G>A, R132H) was most frequent in tumors of the central nervous system, whereas the mutation that we observed in UM (394C>T, R132C) has been identified in several cancers, among which are skin cancers. R132 is the only hotspot observed in IDH1. The F-box and wing domain repeat domain containing 7 gene (*FBXW7*), which has already been implied in UM carcinogenesis [28,29], carried the mutation R479Q in one UM sample. R479 was one of three mutation hotspots in this gene that was mutated in 184 COSMIC cases, 136 of which were R479Q, a mutation found in several cancers but not melanoma. Mutations affecting R183 constituted the major hotspot in the protein phosphatase 2 scaffold subunit A alpha gene (PPP2R1A) found in 120 cases, 38 of which corresponded to the R183Q mutation found in one case of UM. The SWItch/Sucrose Non-Fermentable (SWI/SNF)-related, matrix-associated, actin-dependent regulator of chromatin, subfamily A, member 4 gene (*SMARCA4*) carried an unusual high number of hotspots, with T910 being the amino acid most frequently affected (38 cases). The mutation observed in one UM case, T910M, accounted for 34 of these cases. It was found that 10 of the 31 potential tumor suppressor genes that were mutated in a single or few UM cases carried protein truncating (frameshift or stop gain) mutations compatible with a putative functional consequence. RB1 carried truncating mutation in five UM cases and *BRCA2* in two UM cases (the *BRCA1* mutation did not lead to a truncated protein). Unfortunately, we could not exclude sequencing artefacts which, nonetheless, are very unlikely for oncogenic hotspot mutations.

### 2.2. Expression Analysis

**Figure 2:**
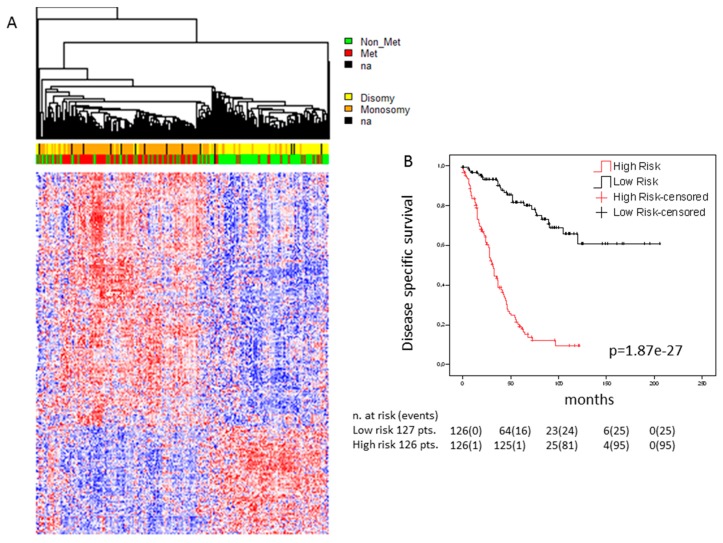
Association of the expression of genes carrying secondary mutations with survival: (**a**) Heatmap of genes carrying secondary mutations whose expression is significantly associated with survival in the cohort of 253 UM cases. Genes were selected applying the bootstrapping algorithm “significance analysis of microarray” with false discovery rate of “0”. Significant genes were ordered by hierarchical clustering (Euclidean distance, average linkage). Gene expression is indicated by a color code according to mean values (red = above mean, blue = below mean, white = mean), the intensity of the coloring indicates the distance from mean. Patient status and chromosome 3 status are indicated in the bars above the heatmap (red = metastatic, green = non metastatic, yellow = disomy, orange = monosomy, black = data not available); (**b**) Kaplan–Meier survival curves. A multigene score (MGS) was calculated by Cox proportional hazard multiple regression model and genes that were independently associated with survival were selected in backward manner. High and low risk was defined by median values of the MGS. Censored cases were lost to follow-up at the time points indicated. Disease-specific survival is shown over time in months.

In order to be functionally involved in tumor development, a gene with oncogenic function must be expressed and a tumor suppressor gene must be or have been expressed. In both cases, expression in a considerable portion of UM cases is expected. We therefore analyzed whether the genes that carry secondary mutations are expressed in UM and, if so, whether their expression is associated with metastatic risk. This analysis was performed on a combined dataset of 253 cases that was cleaned from batch effects as described in the methods section. Most of these genes were seen to be expressed in at least a subset of UM cases. A total of 407 of these genes were significantly differentially expressed in UM cases that developed metastases as compared to those that did not (Figure 2a), 285 genes were up-regulated and 122 genes were down-regulated in high risk cases (Supplementary Table S1). Multivariate Cox regression and Kaplan–Meier survival analysis revealed a subset of 26 genes whose expression was strongly and independently associated with relapse free survival (*p* = 1.87^−27^; Figure 2b). A similar analysis using only the genes annotated as belonging to the calcium signaling pathway or genes annotated in the category of “cancer pathways” yielded similar results (Supplementary Figure S1b,c). These data indicate that the genes that carry secondary mutations might be directly involved in the metastatic process, either through the mutation, through differential expression, or through both. However, we cannot rule out the possibility that at least some of the differential expression events are secondary consequences of dysregulation of other UM-associated driver genes.

### 2.3. PTK2B as a Secondary Driver Mutation

**Figure 3:**
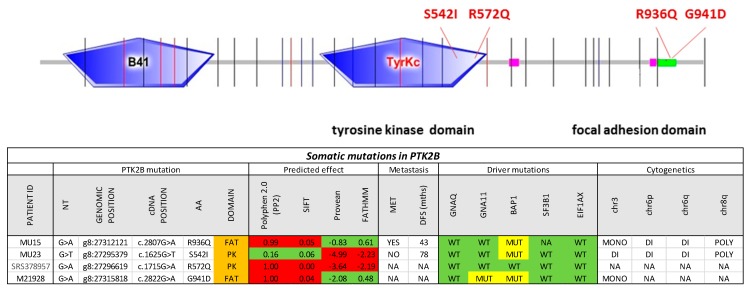
Protein tyrosine kinase 2 beta (*PTK2B*) mutations in uveal melanoma. The entire protein coding sequence of PTK2B is shown. Exon borders are indicated by vertical lines. B41, tyrosine kinase, and focal adhesion kinase domains are indicated by boxes. The table at the bottom shows the characteristics of the mutations, polyphen 2.0, SIFT, PROVEAN, and FATHMM scores, as well as the sample characteristics with regards to known driver mutations and cytogenetic copy number alterations.

In the search of additional potential initiator mutations in GNAQ/GNA11 wildtype tumors, we focused on the protein tyrosine kinase 2 beta (PTK2B) gene given the central role of PTK2B in many intracellular signaling cascades including mitogen activated (MAP)-kinase signaling [30] and the occurrence of mutations in CM including a hotspot in G414 [31,32,33]. In the dataset of Martin et al. [11], we identified a G941D mutation in the PTK2B gene. The mutation was located in the focal adhesion targeting (FAT) region. It occurred in a case with a GNA11 mutation. In the dataset of Harbour et al. [9] there was an R572Q substitution in the tyrosine protein kinase domain of PTK2B in the absence of a GNAQ or GNA11 mutation. We sequenced the region encoding the kinase and the FAT domains by Sanger sequencing in 9 cell lines and an additional 42 cases of GNAQ/GNA11 double wildtype UM from our collection. We found two mutations in two cases, one, R936Q, in the FAT domain and the other, S542I, in the kinase domain (Figure 3). In silico prediction of the effects of these mutations using the algorithms polyphen [34], Sorting Intolerant From Tolerant (SIFT) [35], Protein Variation Effect Analyzer (PROVEAN) [36], and Functional Analysis through Hidden Markov Models (FATHMM) [37] showed high likelihood of functional consequences predicted for the R572Q mutation by all four algorithms, for the S542I by PROVEAN and FATHMM and for the G941D and R936Q mutations by polyphen and SIFT. Allele frequency was compatible with heterozygosity and full penetrance for the two mutations detected by Sanger sequencing, allele frequency was >20% for the samples from the published dataset [10]. None of the cell lines tested carried a *PTK2B* mutation consistent with the low frequency observed in tumors. Further analyses using the ENSEMBL Variant Effect Predictor (https://www.ensembl.org/info/docs/tools/vep/index.html) did not reveal further indications of pathogenicity (Table S4). 

### 2.4. Signatures of Somatic Mutations in UM 

**Figure 4:**
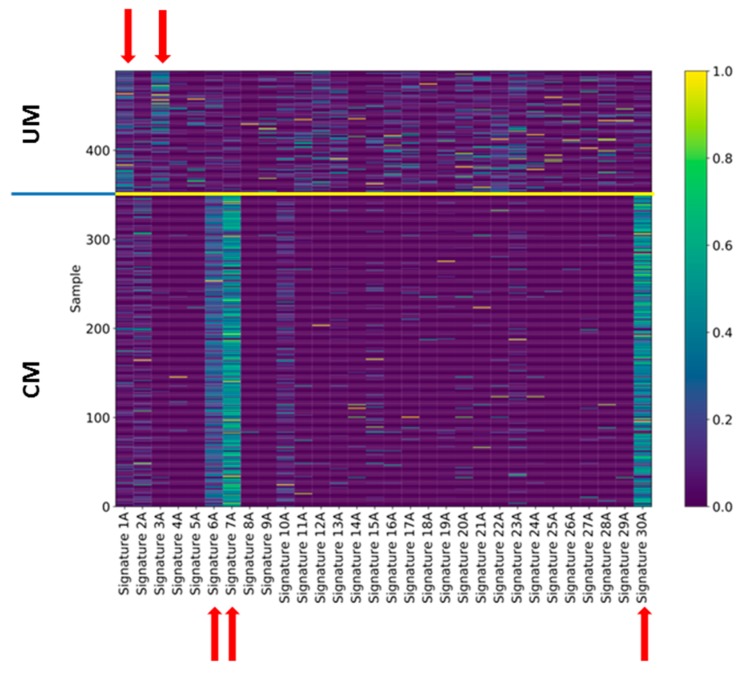
Exposures of uveal and cutaneous melanoma to Alexandrov’s signatures. The exposure of each single case of uveal (upper part) and cutaneous (lower part) melanoma to Alexandrov’s signatures was indicated by a color code (0 = completely absent, 1 = perfect match). The signatures that show the main exposures are indicated by red arrows.

We considered the 139 UM samples for the analysis of mutation signatures. For comparison, we also analyzed 351 TCGA CM samples that show the driver mutations BRAF or NRAS. For each sample, we extracted the related mutational profile and computed Alexandrov’s signatures (A signatures) [16,17] and ad hoc signatures (B signatures) obtained through a novel regularized dictionary learning method [38] outlined in the methods section. We determined the occurrence (exposures) of the 30 mutational A signatures in the single UM samples using the sparse coding method (see methods section). For each signature, we counted how many times it appears in a genome (Figure 4) after discarding the signatures that have a normalized exposure in less than 25% of the cases. For UM (upper panel), signatures 1A and 3A were the most frequent signatures that occurred in 37 and 28 cases, respectively, whereas for CM, the most frequently occurring signatures were signatures 6, 7, and 30. Supplementary Figure S2a,b shows the occurrence of each signature in the CM and UM datasets, respectively. 

For UM, signature 1A was characterized by the triplet NCG that mutated to NTG (N = any) and signature 3A, which had no strong mutation profile. These two signatures have also been identified by Robertson et al. [15]. Signature 1A is associated with age and signature 3A with BRCA1/2 mutations [16]. With regard to the CM, associated signature 7 was the signature associated to the UV-light etiology, whereas signature 6 was associated with defective DNA mismatch repair. Signature 30, instead, was not associated to any known etiology. The A signatures exhaustively explained the CM mutational profiles, yet for UM, no signature was clearly associated with the tumor, as evident from Figure 4 where the exposure assignment for UM was relatively weak (see also Supplementary Figure S2).

In order to identify signatures that can better explain the mutational profile in UM, we applied regularized dictionary learning on the same UM and CM somatic mutation datasets, thus providing a new set of mutational signatures (B signatures). For CM, the new signature 1B (Figure S3a) had the highest exposure rate (Figure S3c), yet the B signatures did not explain more of the variability of the CM mutation profile than A signatures (Figure S3b). Signature 1B showed a high correlation with several A signatures, among which were signatures 6A, 7A, and 30A (Figure S3d). 

**Figure 5:**
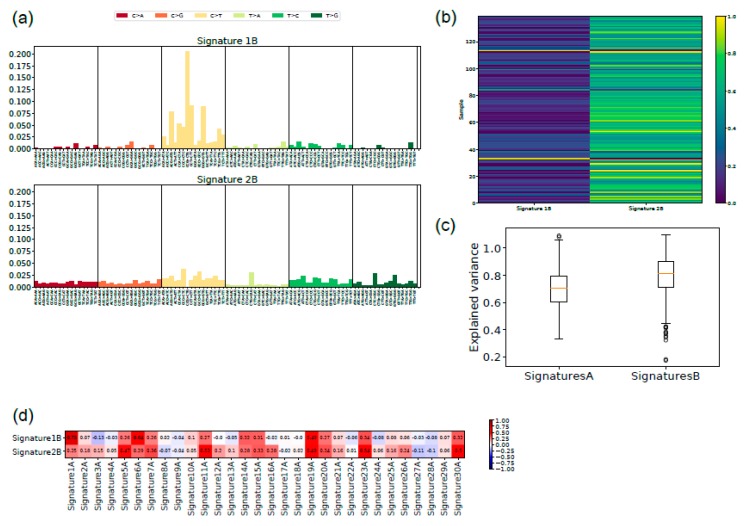
New signatures for uveal melanoma: (**a**) the two signatures with the main exposure for uveal melanoma are shown with the indication of the 96 triplets containing the mutation and the nucleotide 5′ and 3′ to it; (**b**) the explained variance is shown for Alexandrov’s signatures (A) and the new signatures (B). The lower level of the box indicates the 25th percentile, the upper margin indicates the 75th percentile, the red line indicates the mean; (**c**) the exposure of each single case of uveal melanoma to signatures 1B and 2B is indicated by a color code (0 = completely absent, 1 = perfect match); (**d**) correlation of signatures 1B and 2B with Alexandrov’s signatures. Correlation is indicated by a color code (intense red = 1, intense blue = −1, white = 0).

UM signature 1B showed a prevalence of C>T mutations again in the context of NCG, yet now also including the triplets CCN (Figure 5a). Signature 2B, similar to signature 3A, presented T>N mutations in addition to C>T mutations. Most UM cases showed exposure to one of these two signatures (Figure 5c), which explained more of the variability than A signatures (Figure 5b). Signature 2B showed a high correlation with several A signatures, most strongly with signature 1A (Figure 5d). 

We also analyzed the two tumor datasets together to investigate possible signatures shared by the samples (*U* signatures; results are shown in Figure S4). Again, the new signatures explained the observed variance to a higher extent (Figure S4b). The set of exposures was clearly distinct between UM and CM samples (Figure S4c), suggesting different etiological backgrounds for these tumors. The correlation between the unified UM–CM signatures with the single signatures showed that the unified signature 2U correlated with the UM typical signature, and that the unified signature 1U correlated with the CM signature (Figure S4e,f). The comparison with A signatures showed, as expected, correlation of signature 1U with CM-A signatures and of signature 2U with UM-A signatures (Figure S4f).

## 3. Discussion

Tumor evolution is a Darwinian selection process where cells that carry somatic mutations are positively selected because they determine a net growth advantage (more proliferation and/or less cell death). Somatic mutations frequently occur due to environmental insults, such as UV-light for CM or cigarette smoke for lung cancer. Some cancers show genomic instability leading to the accumulation of a high mutational burden. In both cases, in addition to few positively selected mutations that drive tumorigenesis, there are many other mutations that do not affect the viability of tumor cells, the so-called passenger mutations. Mutations that negatively affect tumor growth are negatively selected. Net tumor growth results from the balance between growth promoting (driver) mutations and growth limiting negative mutations [39,40]. 

Driver and passenger mutations are identified operatively—driver mutations occur early during carcinogenesis and are therefore present in most if not all cells forming a tumor, and they occur in many tumors of the same type. Driver mutations normally are hotspot missense mutations in oncogenes or truncating or frameshift mutations in tumor suppressor genes [24]. Passenger mutations are thought of as neutral bystanders that do not affect the fitness of the tumor cell, are infrequent, and often occur in a subclonal manner [40,41]. Most likely, there is a grey zone of somatic mutations that slightly affect tumor fitness, and the cumulative effect of these mutations is linked to the vastly heterogeneous development paths observed for natural human tumors [40,41,42]. 

Since the formulation of the two hits hypothesis by Knudson, one assumes that transformation of a normal cell into a cancer cell needs at least two irreversible steps [43,44], usually mutations in oncogenes and tumor suppressor genes. This concept has later been integrated into the multistep carcinogenesis model that predicts distinct morphological changes during tumor development on the basis of specific molecular lesions [45]. More recently, these models have been integrated into a cancer evolution model distinguished by functional hallmarks [46,47], and molecular analyses of highly heterogeneous colorectal cancers have delivered evidence that many tumors undergo an initial phase of high genomic instability followed by outgrowth of stabilized clones [21,48]. The same tumor evolution path also appears to apply to UM [10], although a phase of genome instability has not been shown for UM. 

Here, we report on the observation that many secondary mutations occur in the same G-protein-related pathways as the known drivers of UM carcinogenesis. The main limitation of the study is that we worked on next generation sequencing data and could not validate single mutations by Sanger sequencing. Sequencing artefacts can occur non-randomly in specific genes, yet it is unlikely that the pathway annotations of these genes are enriched. Given this noise, the enrichment observed will hardly overestimate the real enrichment. Enrichment is also limited due to imperfect annotation. The function of several genes that carry secondary mutations hints at a function in G-protein signaling, although they are not annotated accordingly.

UM has a very low mutational burden, and we showed that the KEGG pathway annotations of mutations other than the few known drivers were significantly enriched in the same pathways that involve *GNAQ* and *GNA11*, especially the calcium-signaling pathway. This might indicate that a single mutation in the pathway, whose aberrant activity drives tumorigenesis, is probably not sufficient to obtain full transformation. We therefore postulate that a second mutation that further destabilizes the control of the pathway is apparently needed, or at least one that accelerates tumorigenesis. According to this hypothesis, mutations in *GNAQ*, *GNA11*, *CYSLTR2*, and *PLCB4* (and the frequently mutated genes *SF3B1*/*SRSF2* and *EIF1AX*) are not sufficient to yield a UM. This is in apparent contrast with data showing that *GNAQ* [5] and *GNA11* [6] mutations, as well as mutations in *CYSLTR2* [14], when overexpressed from transgenic overexpression constructs in spontaneously immortalized melan-A cells, induce tumorigenesis in syngeneic mice. It is unknown whether these drivers need additional mutations to do so. In CM, mutation of *BRAF* is not sufficient for full transformation [49] and it is highly likely that experimental tumorigenesis using xenografts with transgene overexpression in mice does not underlie the same restrictions as natural cancer evolution in humans [50]. Indeed, despite the fact that two hits are sufficient for transformation in experimental melanomagenesis [49], human tumors generally show many more mutations that might affect carcinogenesis driven by mutations in strong driver genes. Relative resistance of the cell towards molecular lesions affecting a growth pathway makes biological sense, inasmuch as it would lower the otherwise high probability of transformation. As the R183 and Q209 mutations of *GNAQ* and *GNA11* have so far not been found in the germline, they might be incompatible with life. There are, however, mosaic cases of *GNAQ* R183 mutations that give rise to port wine stains and Sturge–Weber syndrome, characterized by vascular malformations that, in the latter case, are associated with cerebral symptoms and glaucoma but apparently not with an increased risk of UM [50]. The lack of the association of mosaic *GNAQ* mutations with UM is compatible with the concept of these mutations not being sufficient for neoplastic transformation. 

Our analysis sheds some doubt on *PLCB4* being a true initiating mutation, as in the dataset used, only two of the three different mutations occurred in a case without a *GNAQ* or *GNA11* mutation. In addition to the three mutations in PLCB4, there were three mutations in PLCB1 and five in PLCB2, which was more consistent with our concept of secondary rather than primary driver mutations. A recent report showed, however, the presence of *PLCB4* mutations in melanocytosis that apparently drove the development of UM [51]. 

We selected one of the genes that carried secondary mutations, *PTK2B*, for further analysis. Focal kinases are especially interesting for UM, as they are activated by *GNAQ* and their inhibition inhibits Yes-associated protein (*YAP*) signaling and UM growth [52]. The relatively rare mutations in *PTK2B* and their distribution are consistent with its activity as a secondary driver with tumor suppressor activity, as described for breast cancer [53], or as an oncogene, as described for hepatocellular carcinoma [54]. We found two more mutations by sequencing 42 cases. Only one of the mutations in the tyrosine kinase domain was predicted to alter protein function by all four algorithms and no other evidence of pathogenicity could be collected. No case showed the hotspot mutation found in several cases of CM [31,32]. It was unclear whether these mutations contributed to tumor development.

We applied regularized dictionary learning [38,55] on the 139 UM cases, which led to the identification of two new mutational signatures and the corresponding set of exposures. The two signatures identified were fairly correlated to signatures identified when applying Alexandrov’s algorithm, yet the new signatures, especially signature 1B, were more informative for UM than Alexandrov’s signatures. This was apparently due to the fact that the main consensus of NCG was expanded to NCG and CCN. Light exposure and iris color have been associated with different *GNAQ* and *GNA11* mutations, although they affected the same nucleotide [56]. However, the type of mutations in these driver genes, A>T or A>C, did not correspond to the UM-specific signatures, just as *BRAF* mutations in CM did not correspond to the UV mutation signature. The comparison between UM and CM signatures delivers evidence for a different etiology for the two tumors, UV light for CM and an unknown etiological factor for UM, consistent with the fact that the vitreous body and the lens absorb most of the UV radiation [57].

## 4. Materials and Methods 

Samples of UM were obtained from enucleated eyes with informed consent form the patients within the project GALL12-2011, with amendment 01–10/05/2016 approved by the Regional Bioethics Committee. Samples were treated and DNA was extracted from samples conserved in RNAlater or formalin-fixed paraffin-embedded tissues, as described [58]. 

Mutation data were imported from the analysis by Field et al. [10]. All mutations with the exception for the known putative driver mutations (*GNAQ*, *GNA11*, *CYSLTR2*, *PLCB4*, *SF3B1*, *SRSF2*, *EIF1AX*, *BAP1*) were collected and analyzed together for potential enrichment of KEGG pathway annotations using EnrichR [59]. 

We used data derived from three cohorts of primary UM for a total of 333 cases (124 from the Department of Ophthalmology, Leiden University Medical Center, Leiden, The Netherlands [60]; 66 from the Laboratory of Molecular Pathology, IRCCS Ospedale Policlinico San Martino, Genoa, Italy [58,60]; 63 from Institut Curie, Paris, France [61]; and 80 from The Cancer Genome Atlas – uveal melanoma (TCGA-UVM) dataset (http://cancergenome.nih.gov/) [15]). For patient/sample characteristics, see Table S5. 

The combined dataset contained 345 cases. Chromosome 3 status was known for 276 cases, clinical follow-up was available for 333 cases, and somatic mutations were known for 221 cases. For 253 of these cases, gene expression profiles were available (GSE27831, GSE51880, GSE22138, TCGA-UVM).

Gene expression analyses were performed in R (https://www.r-project.org/). For gene expression analysis, microarray probes were collapsed to gene symbol to the maximum variance probe set (WGCNA package [62]). The expression profiles were merged into a single dataset after batch effects removal using the Combating Batch Effects When Combining Batches of Gene Expression Microarray Data (COMBAT) algorithm [63] implemented in the inSilicoMerging package [64]. TCGA-UVM gene expression data were obtained from UCSC-Xenabrowser (http://xena.ucsc.edu/; level 3 data, log2 (x+1) transformed RNA-Seq by Expectation Maximization (RSEM) normalized counts). Gene selection analyses were performed as previously described [60,65,66,67]. Kaplan–Meier survival analyses and log rank (Matnel–Cox) were performed as previously described [60].

Statistical analyses for gene expression analyses were performed using Significance Analysis of Microarray (samr package) [68] as previously described [69].

The metastatic risk by Kaplan–Meier survival analysis and COX proportional hazard multiple regression model was tested as previously described [60,69]. A multigene score was calculated with backward method for the selection of the genes that are significantly differentially expressed between low and high risk patients (removing the non-significant variables sequentially). Disease-specific survival and distant metastasis were used as endpoints. *p*-values refer to Student’s *t*-test or chi-square, as indicated in the legends. Differences with a *p*-value < 0.05 were considered statistically significant. 

Mutational signatures are patterns of somatic mutations that are characteristic for environmental or biological factors that can lead to cancer pathogenesis. If we did not consider the information about DNA strand, there were six possible substitutions that could appear in the genome: C>T, C>G, C>A, T>C, T>A, T>G. In order to enrich this representation, these substitutions were considered in the context of the nucleotides immediately in the 5’ and 3’ positions with respect to the mutated base, resulting in a list of 96 unique triplets each containing a mutation. These vectors can be expressed as a matrix X∈ R139 × 96.

Alexandrov et al. [16] showed the efficacy of the application of a methodology based on matrix decomposition [17] for the extraction of mutational signatures and corresponding exposures, which define how relevant the signatures are for each given genome [16]. The current literature accounts for 30 signatures that have been extracted from different types of cancer [16]. 

We used the code provided in the R package decompTumor2Sig [70] to determine the exposures of these 30 signatures in the UM somatic mutation data, following a sparse coding approach. The resulting exposures were thresholded to delete all the signatures that had a contribution less than 0.25% for each genome, as suggested in [17]. 

We performed a refinement of the method used by Alexandrov solving a problem of the form:

$$\begin{array}{l} S^{*},\;C^{*}=argmin{\left\|X-CS\right\|}_{F}^{2}+\lambda_{1\;}{\left\|C\right\|}_{1}\;+\lambda_{2}\;{\left\|S\right\|}_{1\;}\\ \;S,C \end{array}$$

where ***X*** is the data matrix, ***C*** is the exposures matrix, and ***S*** is the signatures matrix. This approach, regularized dictionary learning, guarantees a better solution in terms of robustness to noise and reconstruction error [37,54]. 

Three hyper-parameters must be tuned: the number of signatures, λ_1_ and λ_2_. The tuning of such hyper-parameters was done by consistency analysis, by optimizing the problem with a random initialization of the matrix C multiple times. The parameter k was selected in the set [2,4,6,8,10] while λ_1_ and λ_2_ were chosen by random search in the interval (1 × 10^−5^,1 × 10^−2^) (see Supplementary). The final solution was obtained by initializing the matrix C in a deterministic fashion on the basis of an singular vector decomposition (SVD)-based procedure [54,71,72]. The code can be obtained at: https://github.com/veronicatozzo/dalila.

PTK2B exons 19–27 and 34–36 to be sequenced were amplified by polymerase chain reaction using 25 ng of genomic DNA in a 25 μL reaction mix including 10× Platinum PCR Supermix, 1.5 mM MgCl_2_, 200 μM deoxy-nucleotride triphosphates (dNTPs), 1 μM primers, and 0.5 U Taq Platinum (Invitrogen—Life Technologies Corporation, Milan, Italy) on a Veriti 96-well Thermal Cycler (Applied Biosystems—Life Technologies Corporation). Thermal cycling conditions were as follows: 94 °C for 4 min, followed by 25 cycles of 94 °C for 30 s, 58 °C for 30 s, 72 °C for 30 s, and a final extension at 72 °C for 7 min. All PCR primers (see Table S6) were designed with a universal sequence at 5′-end (Universal Forward primer 5′-GTTGTAAAACGACGGCCAGT-3′) and an M13 (-48) reverse primer 5′- GTGTGAAATTGTTATCCGCT-3’) to perform single-pass sequencing. Mutational screening was carried out by direct sequencing of fragments obtained by PCR using an ABI3730 Genetic Analyzer (Applied Biosystems-Life Technologies Corporation, Italy). Sequencing data were analyzed using SeqScape v2.5 software (Applied Biosystems-Life Technologies Corporation, Italy).

Cell lines analyzed were MEL290, MEL270, MEL285, OCM1, OCM3, OMM1, OMM2.5, UPMM2, and UPMM3.

All sequence analyses were based on GRCh37/hg19 draft assembly of the genome. 

## 5. Conclusions

In conclusion, it is highly likely that secondary mutations affect tumor development and progression. Exon sequencing of UM cases should continue, as larger datasets could likely consolidate the information on secondary drivers and identify primary drivers for the 10–15% that do not carry mutations in the known drivers. The specific combination of primary and secondary drivers is expected to contribute to the heterogeneity of tumor presentation, development, and response to therapy observed in the clinics, and its analysis appears necessary for true personalization of therapy. Secondary driver mutations might determine the observed heterogeneity of MAP-kinase pathway activation in UM [62]. The concept of secondary driver mutations should further be tested for validity across all tumor types. In second generation personalized medicine, drug combinations targeting the main actor and additional secondary drivers could prove superior to one-fits-all drug combinations.

## Figures and Tables

**Table 1:** Enrichment analysis of KEGG pathway annotations for secondary genes with somatic mutations.

KEGG Pathway	# of Query Genes	# Total Annotated	*p*-Value	Adjusted *p*-Value	Genes
Aldosterone synthesis and secretion *	41	98	1.38 × 10^−6^	3.79 × 10^−4^	*SCARB1, CAMK2D, ITPR1, ADCY4, ITPR2, ATP1A3, ADCY3, ATP1A2, CACNA1D, ITPR3, CACNA1C, CALML4, CACNA1F, CACNA1H, LIPE, CACNA1I, MC2R, PRKACG, CACNA1S, CAMK2G, PRKCG, KCNJ5, CAMK1D, HSD3B2, PRKCB, HSD3B1, ATP1B4, ATP2B2, PRKCA, AGT, NR4A2, POMC, PLCB3, AGTR1, PRKD2, CAMK1, PRKD1, PLCB1, DAGLB, PLCB2, ATF4*
Calcium signaling pathway *	66	188	2.48 × 10^−6^	3.79 × 10^−4^	*RYR1, RYR2, CHRM1, ATP2A3, ATP2A1, CALML4, RYR3, MYLK3, SLC8A1, MYLK, HTR6, GRM5, CCKAR, EDNRB, PRKACG, BDKRB1, PLCE1, NOS1, PDGFRB, PRKCG, PDGFRA, CAMK1D, PRKCB, SPHK1, PRKCA, ITPKB, PLCB3, ADORA2A, LTB4R2, ADORA2B, AGTR1, PLCB1, SLC25A5, PLCB2, CAMK2D, PDE1C, CACNA1B, ITPR1, ADCY4, ITPR2, ADCY3, CACNA1D, ITPR3, CACNA1C, CACNA1F, CACNA1E, EGFR, CACNA1H, CACNA1I, GRIN2A, ERBB4, PTK2B, CACNA1S, PLCG1, CAMK2G, NTSR1, NOS2, NOS3, ATP2B2, PHKB, TPCN2, TPCN1, GRIN2D, CAMK1, PLCD4, MCU*
Thyroid hormone signaling pathway	45	116	5.08 × 10^−6^	5.17 × 10^−4^	*NOTCH2, NOTCH3, NOTCH1, HDAC1, ITGB3, SLC2A1, ATP1A3, ATP1A2, MED16, HIF1A, ACTB, SLC9A1, MED17, CASP9, MED12, MED14, MED13, MYC, PRKACG, EP300, PLCE1, PLCG1, SLC16A2, RXRG, PRKCG, NCOA2, CREBBP, PRKCB, ATP1B4, TSC2, SLC16A10, PRKCA, ESR1, MTOR, MED13L, BMP4, KAT2A, PLCB3, PIK3CA, CTNNB1, PLCB1, PLCD4, PLCB2, PFKP, MYH6*
Ras-proximate-1 (*RAP1*) signaling pathway *	67	206	3.69 × 10^−5^	2.87 × 10^−3^	*FLT4, ITGB3, CALML4, SIPA1L3, ACTB, IGF1R, FGF9, KDR, RAC2, RAC3, PLCE1, MAP2K3, MAGI1, PDGFRB, PRKCG, PDGFRA, PRKCB, ARAP3, PRKCA, NGF, VAV2, TIAM1, PLCB3, MRAS, ADORA2A, PIK3CA, ADORA2B, PARD3, KIT, RAPGEF2, PFN4, PRKD2, TLN2, PRKD1, PLCB1, PLCB2, MET, RAPGEF6, RGS14, GNAI3, ADCY4, FPR1, ADCY3, LPAR3, RASGRP2, THBS1, EGFR, GRIN2A, KRIT1, PLCG1, FGF23, NGFR, ANGPT4, EGF, VEGFB, GRIN2B, FGF17, EFNA2, FGF19, CTNNB1, TEK, FGFR4, FGFR2, LAT, SIPA1, FGFR1, FGF10*
Protein digestion and absorption	35	90	5.15 × 10^−5^	3.14 × 10^−3^	*COL17A1, COL18A1, PRSS1, CPB2, COL14A1, COL11A2, SLC1A1, ATP1A3, ATP1A2, SLC8A1, PRSS3, SLC36A1, CPA2, SLC6A19, SLC15A1, COL27A1, KCNJ13, COL22A1, ATP1B4, SLC16A10, COL1A1, SLC9A3, COL3A1, SLC7A7, COL2A1, COL5A1, COL4A2, COL4A1, XPNPEP2, MEP1A, COL5A3, COL5A2, COL4A3, COL9A1, COL9A2*
Cortisol synthesis and secretion	26	65	2.69 × 10^−4^	9.92 × 10^−3^	*SCARB1, ITPR1, ADCY4, ITPR2, ADCY3, CACNA1D, ITPR3, CACNA1C, CACNA1F, CACNA1H, CYP17A1, CACNA1I, MC2R, PRKACG, CACNA1S, PDE8B, PDE8A, HSD3B2, HSD3B1, AGT, POMC, PLCB3, AGTR1, PLCB1, PLCB2, ATF4*
ATP-binding cassette ABC transporters	20	45	2.59 × 10^−4^	9.92 × 10^−3^	*ABCA1, ABCA2, ABCC4, ABCD2, ABCC2, ABCB1, ABCB4, ABCC5, ABCA3, ABCA4, ABCC6, ABCA9, TAP2, ABCA7, ABCA12, ABCB11, ABCA13, ABCA10, ABCB10, ABCD1*
Circadian entrainment *	35	97	2.93 × 10^−4^	9.92 × 10^−3^	*RYR1, GRIA1, RYR2, CAMK2D, GNAI3, ITPR1, ADCY4, ADCY3, CACNA1D, ITPR3, CACNA1C, CALML4, RYR3, CACNA1H, CACNA1I, GRIN2A, RASD1, PRKACG, NOS1, CAMK2G, PRKG1, PRKCG, KCNJ5, PRKCB, PRKCA, GRIN2B, GRIN2D, PER1, PLCB3, GNB2, NOS1AP, GNB1, GNB3, PLCB1, PLCB2*
Cushing syndrome	51	155	2.15 × 10^−4^	9.92 × 10^−3^	*RB1, SCARB1, PRKACG, PDE8B, PDE8A, MEN1, USP8, AXIN1, AIP, PLCB3, AGTR1, PLCB1, PLCB2, ATF4, KMT2D, CAMK2D, KMT2A, TCF7, GNAI3, ITPR1, ADCY4, ITPR2, ADCY3, CACNA1D, ITPR3, CACNA1C, CACNA1F, EGFR, CACNA1H, CYP17A1, CACNA1I, PDE11A, MC2R, RASD1, DVL2, DVL3, CACNA1S, WNT2, CAMK2G, TCF7L2, WNT10A, TCF7L1, FZD2, HSD3B2, FZD5, WNT3A, HSD3B1, AGT, POMC, APC, CTNNB1*

* = pathways that contain G-protein subunit alpha Q (GNAQ) or G-protein subunit alpha 11 (GNA11).

## Supplementary Materials

The following are available online at https://www.mdpi.com/2072-6694/11/11/1688/s1: Figure S1: Kaplan–Meyer survival analysis using the genes carrying secondary mutations that are annotated as belonging to “KEGG Cancer Pathways” or “Calcium Signaling”, Figure S2: Frequency of Alexandrov’s signatures in cutaneous and uveal melanoma, Figure S3: New signatures for cutaneous melanoma. Figure S4: Unified signatures for uveal and cutaneous melanoma, Table S1: Gene lists, Table S2: KEGG pathway annotations for GNAQ, GNA11, CYSLTR2, and PLCB, Table S3: Extended KEGG pathway analysis, Table S4: PTK2B variant effect predictor, Table S5: Patient/sample characteristics, Table S6: Sequencing primers.
