# Supplementary material for: Secondary Somatic Mutations in G-Protein-Related Pathways and Mutation Signatures in Uveal Melanoma
Source: Cancers (Basel). 2019 Oct 30;11(11):1688. doi: 10.3390/cancers11111688 (PMC6896012; doi:10.3390/cancers11111688)
Supplement: Supplementary file 1 [file cancers-11-01688-s001.zip › cancers-548928-Suppl_figures.pdf]

Supplementary Materials:

Secondary Somatic Mutations in G-Protein-Related Pathways and Mutation Signatures in Uveal Melanoma

Francesca Piaggio, Veronica Tozzo, Cinzia Bernardi, Michela Croce, Roberto Puzone, Silvia Viaggi, Serena Patrone, Annalisa Barla, Domenico Coviello, Martine J. Jager, Pieter A. van der Velden, Michael Zeschnigk, Davide Cangelosi, Alessandra Eva, Ulrich Pfeffer and Adriana Amaro

	Chi-Square	df	Sig.
Log Rank (Mantel-Cox)	126.623	1	.000

	Chi-Square	df	Sig.
Log Rank (Mantel-Cox)	61.250	1	.000

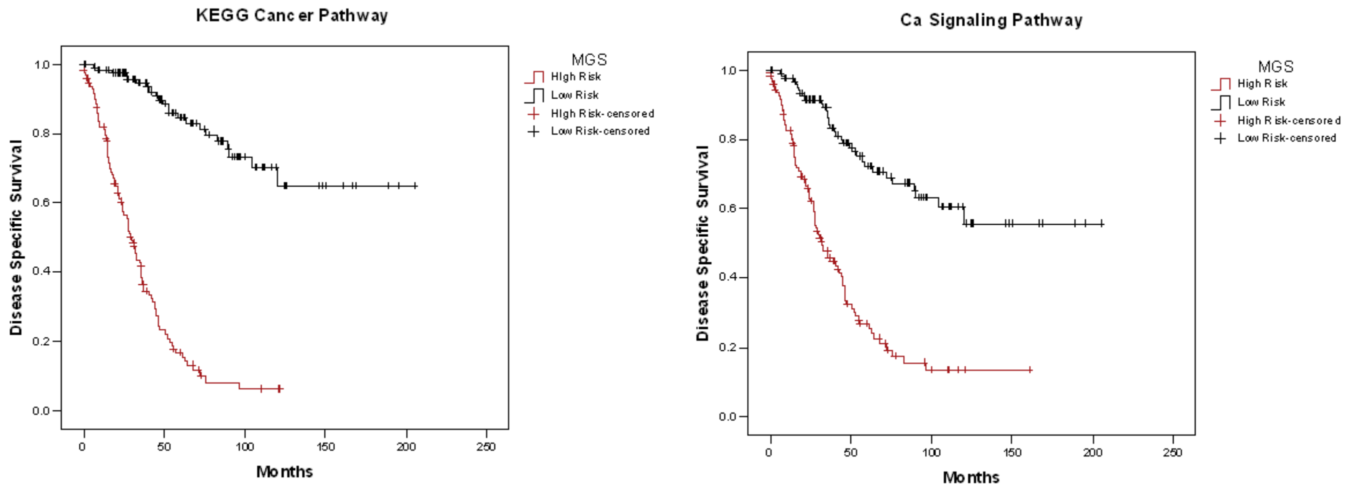

N° at Risk (Events)	0	50	100	150	200
Low Risk 126pz	126(0)	68(11)	25(20)	6(22)	0(22)
High Risk 127pz	126(1)	21(86)	3(98)	0(98)	

N° at Risk (Events)	0	50	100	150	200
Low Risk 127pz	126(0)	62(23)	22(33)	5(34)	0(34)
High Risk 126pz	125(1)	74(27)	5(86)	0(86)	

Figure S1. Kaplan–Meyer survival analysis using the genes carrying secondary mutations that are annotated as belonging to “KEGG Cancer Pathways” or “Calcium Signaling”.

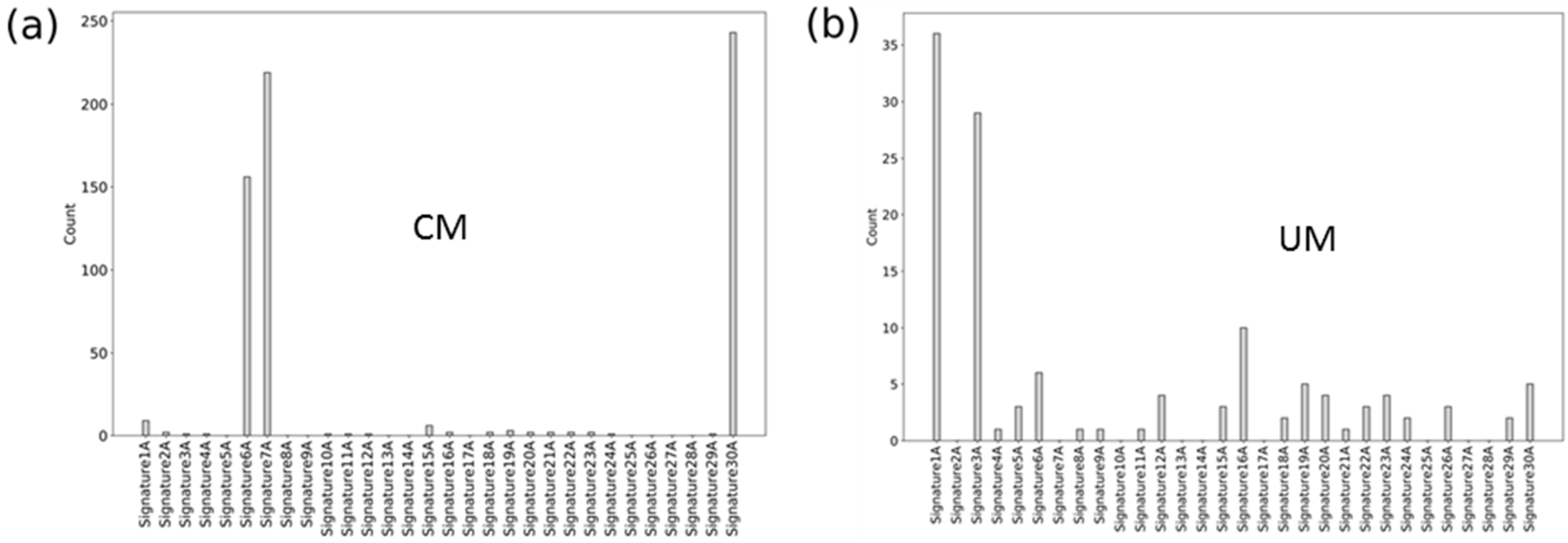

Figure S2. Frequency of Alexandrov's signatures in cutaneous and uveal melanoma.

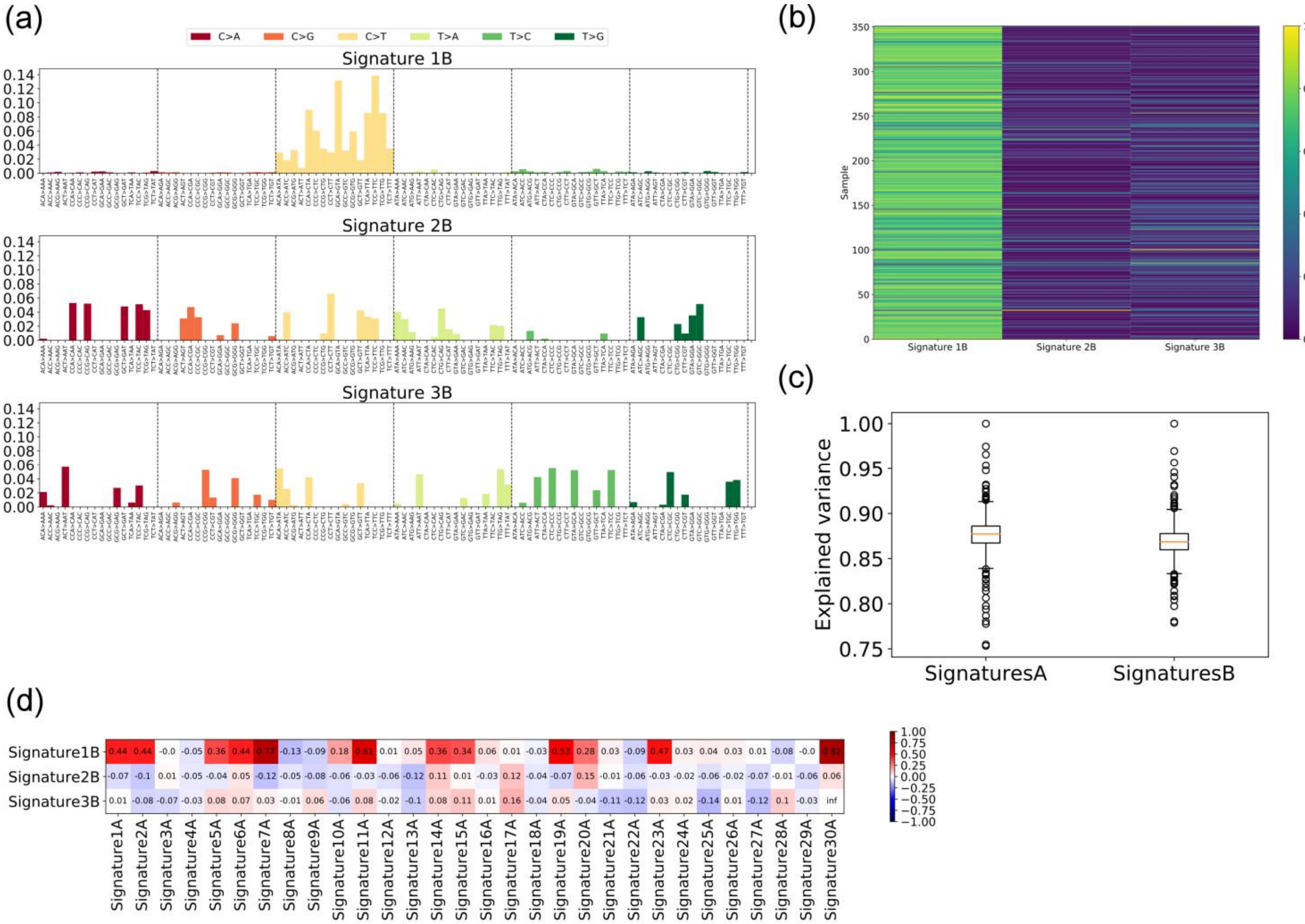

Figure S3. New signatures for cutaneous melanoma.

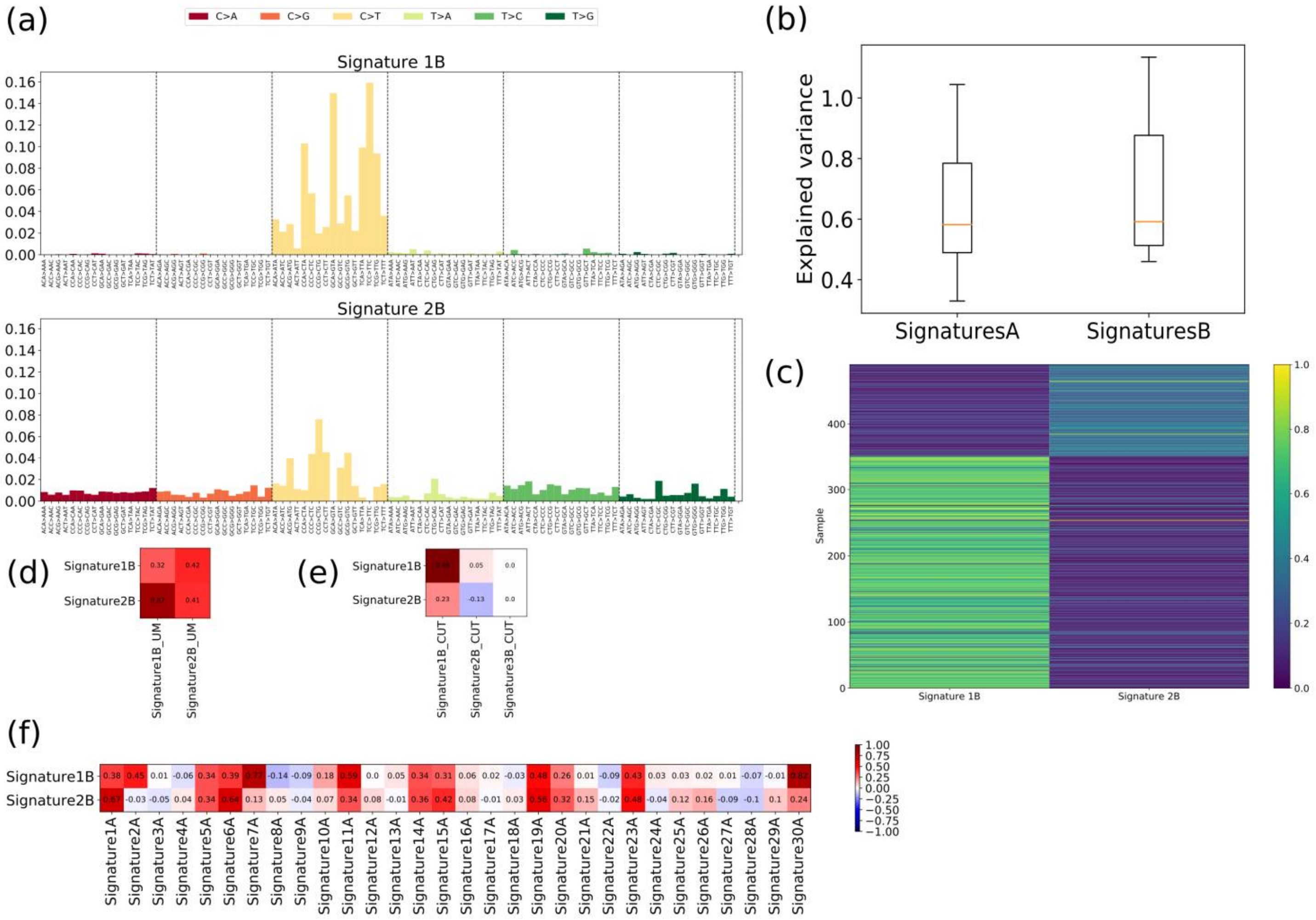

Figure S4. Unified signatures for uveal and cutaneous melanoma.
